# Social Cognitive Theories and Electronic Health Design: Scoping Review

**DOI:** 10.2196/11544

**Published:** 2019-07-19

**Authors:** Patricia Grace-Farfaglia

**Affiliations:** 1 Department of Nutritional Sciences University of Connecticut Waterbury, CT United States

**Keywords:** social theory, design, health promotion, behavioral economics, social support, social media, serious games

## Abstract

**Background:**

There are several social cognitive theories (SCTs) and models that support platform design in electronic health (eHealth) promotion trials. The rationale for this scoping review was to determine how social design features (informational aid, expressive support, gaming, and tailored content) are used to promote self-efficacy, engagement, knowledge, and behavior change.

**Objective:**

This study aimed to review a broad spectrum of digital health interventions in the literature seeking trials that use SCTs for the design of eHealth applications.

**Methods:**

The author conducted a systematic scoping review of 161 Web-based health interventions from published randomized clinical trials using 1 or more tools to address the social cognitive determinants in their website design from January 2006 to April 2016. An iterative approach was used in the selection of studies and data extraction. The studies were analyzed for quality and coded for type of social design features employed.

**Results:**

Expressive interaction tools were found in 48.6% (54/111) of studies categorized as a strong recommendation by the Joanna Briggs Institute criteria. Overall, less than half of the studies addressed participant social support and motivational needs (43.8%). The vast majority of studies (100%) relied on the use of the Web for delivery of informational aid and tailored content for the individual participant (75.9%).

**Conclusions:**

This review fills a research gap by linking social theory to Web strategy to improve the impact and sustainability of eHealth interventions. A Digital Health Intervention Model was developed to provide a framework to enhance future Web-based health intervention design and execution.

## Introduction

### Background

The aim of this scoping review was to review published clinical trials for evidence of social cognitive theory (SCT)-driven design of electronic health (eHealth) interventions. A secondary objective was to review the construct of social support as it applies to Web-based social interactions and recommend the inclusion of tools that increase media social presence to explore its value in the design and measurement of health behavior outcomes [1]. Finally, this paper offers an integrative theoretical perspective and framework for health researchers and designers to engage users to remain digitally connected for better health outcomes.

### Social Theories and Electronic Health Design

Early in the internet era, Bandura envisioned that SCT would function as part of a self-regulatory delivery system for computer-assisted health interventions [2]. Bandura’s work demonstrated that learning takes place within a social context. A 2015 systematic review and meta-analysis of SCT-based interventions for patients with cancer for diet and physical activity (PA) found only 18 articles that met the inclusion criteria of reporting 1 or more SCT constructs in the design [3]. The scarcity of theory-based design studies leaves a wide gap in our understanding of the Web-based experience factors contributing to health behavior change and maintenance [4]. Authors of a recent systematic review developed a taxonomy of 36 social media features and described their use in 134 studies, reporting that the majority reported positive effects including engagement, satisfaction, usefulness, social support, and behavior change (70%) [5]. However, the remainder of the papers reported no behavioral change (28%) or negative outcomes (2%). These findings suggest that social influence tools in digital behavior change interventions may produce unintended effects.

SCT is an integrated model of *emergent interactive agency* where personal factors and environmental events function as interacting dependent variables and operate as reciprocal factors predicting behavior. This theoretical framework has greater saliency today, as more consumers digitally track their health behaviors and are connected to external interactive guidance and social support [6]. In SCT, the constructs of self-observation, judgmental process, and self-reaction comprise a system of self-regulation of motivation and behavioral action. However, too often in the studies of Web-supported interventions reviewed for this paper, social interactions took second place to techniques that offered information, monitoring, and self-management tools [7].

SCTs, such as the health belief model, theory of reasoned action, and theory of planned behavior recognize the role of social support as an important determinant of health behavior. But it is essential that health researchers define the type of social support sought and received by the user to guide their study design and predict how social presence will perform within the digital medium before study implementation. The dimensions of Web-based social support have been classified as instrumental, socioemotional, and informational [8]. The digital environment gives ready access to asynchronous, simultaneous, and bidirectional social support.

A survey of 240 health-related websites rated the quality of social media tools and use of evidence-based theory for Web design [9]. Quality was determined by the presence of behavioral components, interactivity, and user-generated content in the design. Nearly half of the sites offered feedback, which consisted primarily of simple guidelines rather than tailored advice. The primary applications for content sharing were status updates, discussion forums, sharing success stories, sharing photos blogs, and comments. Overall, reviewers gave low marks to the sites as they lacked tools that promote theory-based behavior change.

### Social Presence and Personalization Impact Behavior Change

Social presence, the perception of *nonmediation*, conveys the sensation of intimacy and immediacy in digital communication [10]. Studies comparing real-world face-to-face with digital discussions in health interventions have found that they promote adherence and behavior change [11,12]. As observed in studies of interactions of teens on Web-based social network , teens engage more fully when they cocreate content and develop Web-based self-identities through emojis, video, and other audiovisual materials [13] contributing to the feeling of being copresent with peers within a virtual environment [14]. Adult focus groups of users of a Web-based social network for health behavior change suggested to researchers that the addition of personalization options such as pictures, recipes, and status updates for social interaction and comparison are desirable options [15]. The quality of social presence is unique to each media channel; therefore, choice of medium has a direct effect on the depth of information processing and user motivation to take greater effort to process a message. Iterative health information sharing by social media users has resulted in many benefits including enhanced self-efficacy and healthy lifestyle adoption in multiple studies [16].

**Figure 1:**
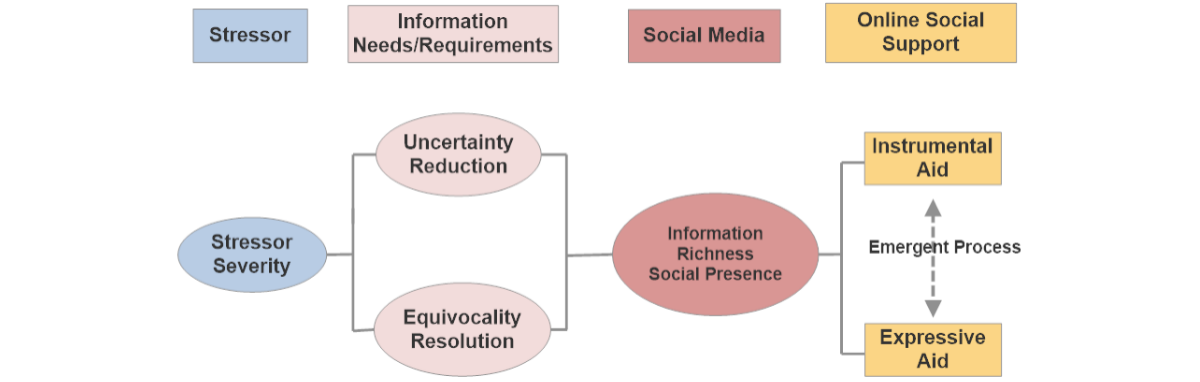
Social media and social support.

When faced with uncertainty, humans have higher information needs and will seek out trusted sources of information. Lee and Kvasny (2013) proposed that information richness and social presence of the Web experience satisfy the needs of an individual to obtain instrumental and expressive support [8]. Figure 1 displays a theoretical framework of how social media use and Web-based social support address these needs. This model illustrates that uncertainty is a consequence of a person’s self-appraisal of efficacy. The reduction of an uncomfortable state of uncertainty motivates the health consumer to seek out experts or Web-based peers.

As communities of caring have proliferated on the Web, so has the ability to access meaningful solutions for health problems. Social connections can be established based on similarities and differences important to the Web user and can be established through member profiles or disease-focused forums. An overview of the social media, social presence, and information richness characteristics of different websites is shown in Table 1 [8,17-19].

**Table 1:** Social media characteristics.

Social media form		Information richness	Social presence	Social Support Scale 1-6
**Blogger or WordPress editable pages or wikis such as**				
	Wikipedia	Essays and short reports via blogs	Blogs; narrative or storytelling	1-2
	Google Docs			
Audio sharing through Clyp or SoundCloud		Music and voice	Collective experience and content community	4
**Videos and collaborative spaces distributed through services**				
	Pinterest	Music, voice, video, collaboration, and reviews	Collective experience and content community	4
	YouTube			
	Vimeo			
	Viddy			
**Combined, multipurpose platforms that offer multiple media options**				
	Facebook	Essays, short reports, and links to articles	Social network; pictures; videos; messaging; leaders and followers	5
	Ning			
	LinkedIn			
	Skype			
**Short-form text messaging and photo sharing**				
	Twitter	Texting: music, movies, books, maps, voice, high quality photos, and videos	Social network; pictures; immediacy; reach; leaders and followers	5
	Instagram			
	Snapchat			
	Gtalk			
**Mobile health (mHealth) apps**				
	eHealth record	Practice of public health and medicine through mobile devices	Collective experience; content community; information; motivation; support; remote monitoring; diagnostics and decision support	6
	Social health			
	Web-based health communities			
	Fitness apps			
	Personally controlled health management systems (PCHMS)			
**Gamification (serious health gaming)**				
	Multiuser Dungeons (MUD)	Virtual social worlds with identities and collaborative content; challenge; competition; avatars	Collective experience; content community; shared emotional states; social network; social influence	6
	Re-Mission2			
	DietBet			

### Scoping Review of Social Cognitive Theories in Electronic Health Design

This scoping review will summarize various SCTs and digital methods used within a wide spectrum of digital health interventions. During the planning of this paper, it was clear that early eHealth trials too often neglect to report a theoretical basis for their research design. Thus, the initial keyword search criteria yielded only 4 usable trials. With the 2010 publication of Consolidated Standards of Reporting Trials of Electronic and Mobile HEalth Applications and onLine TeleHealth (CONSORT-EHEALTH) guidelines, authors began specifying the mode of delivery and type of trial in the title of the paper, along with study details that would facilitate search retrieval [20].

## Methods

### Overview

Keywords were chosen to locate papers with the characteristics and tools of Web-enabled health intervention within a randomized controlled trial (RCT) or cohort study based on social cognitive or learning theories that informed study design. The researcher used a coding scheme based on the work of Lee and Kvasny with the addition of serious gaming to summarize the results so that research gaps and opportunities could be identified [8]. Using the guidelines of the Joanna Briggs Institute (JBI) manual for the levels of evidence and grades of recommendations and the Preferred Reporting Items for Systematic reviews and Meta-Analyses extension for Scoping Reviews (PRISMA-ScR), papers meeting the criteria for inclusion were graded and summarized [5,21].

### Search Strategy

The University of Connecticut Library databases (PsychInfo, Cumulative Index to Nursing and Allied Health Literature [CINAHL], EBSCO), ProQuest, and PubMed were thoroughly searched using the search terms internet, Web, health, intervention, social presence, expressive aid, instrumental aid, information richness, and design for the years 2006 to 2016.

#### Intervention Criteria

Any health promotion or guidance designed to influence health behavior was based on SCT [6] or explicitly described and referenced any SCT component (such as *self-efficacy*):

- Comparator: any parallel control group.
- Study design: RCTs or cohort.

A total of 399 studies were identified by key word search alone. Using CrossRef citations for the CONSORT-EHEALTH guidelines, 146 additional studies were identified as of April 2016. A total of 161 studies met the RCTs risk of bias and study quality [22,23]. Multimedia Appendix 1 outlines the PRISMA flowchart of the study selection process.

The trials retrieved were coded by theoretical framework, presence of informational and expressive support in the study design, and use of tailored content. Identification and coding of the theoretical framework was hindered by the need to cross-reference previously published papers identified by the authors as the basis for their protocol or multiple papers from the same study data. If the study design was consistent with the outcomes of that framework, it was retained. The most common model for tailoring the Web intervention was social-cognitive learning theory (N=106). Several studies identified therapeutic frameworks adapted for the Web, such as internet cognitive behavioral therapy (iCBT) [24]. The use of the iChange model for health behavior which used wearable measurement tools is a growing trend to facilitate lifestyle change by giving feedback to the participant [25-27]. Gamification, the use of games used seriously (GUS), and fun theory were successfully used to foster participation in 4 studies, and a recent meta-analysis of trials using games for health behavior change reported significant positive outcomes in 9 out of the 10 studies meeting their criteria [28-31]. The use of fun theory was uniquely used in serious video gaming and physical therapy where adherence to the daily practice of mundane tasks is fostered by a challenge and excitement [32,33].

A qualitative analysis of this literature was undertaken because of the diversity in methodologies. The highest quality evidence followed the CONSORT-EHEALTH or TREND guidelines, although a few quality trials predated the guidelines, and recent meta-analyses were included in the discussion [20,34,35].

## Results

### Overview

The literature search retrieved 447 results from PubMed (n=296), University of Connecticut Library databases (PsychInfo, EBSCO, and CINAHL; n=72), and ProQuest (n=79). After removal of duplicates (n=48), 399 titles and abstracts were screened, 215 full-text articles were reviewed, 4 were excluded as additional qualitative papers from a mixed-methods design trial, and 161 articles met the selection criteria (Multimedia Appendix 1). Upon further inspection, there were 9 papers that were overlapping manuscripts from the same dataset which brought the final total to 152 studies. Studies were coded by the author for theoretical framework, tools (informational aid, expressive aid, and gaming), and content that was tailored to each user (Multimedia Appendix 2 [27,36-196]). From this process, the author evaluated different theoretical frameworks and the use of Web intervention tools and environments to promote desired health outcomes. The JBI levels of evidence and grades of recommendations were used to evaluate the studies [187].

### Risk Preference and Goal Setting

Setting personal health goals and evaluating one’s ability to attain them is an essential part of chronic disease management. It has been observed in behavioral economics that loss aversion is a powerful motivator, and this trait is important in setting personal goals. It has been observed that obese individuals are more likely to be risk-seeking, rather than risk-averse, when making decisions that offer uncertain options, which is a tendency related to impulsivity. In Prospect Theory, this is known as risk preference [188,189]. One study observed participant risk taking behavior by allowing participants to bet on the outcome of a weight loss challenge on a commercial website, DietBet [190]. The researchers theorized that offering frequent and small incentives on a social gaming site would influence players to lose weight. Members placed a bet and joined a social game where they wagered that they could achieve the goal of losing 4% of their total body weight and reported their progress to other participants. The financial and social influencers were effective in supporting weight loss as measured by self-reported weight, bets placed, frequency of social interactions, and weigh-in reports on Facebook. A month-long study of the effect of financial incentives within a social GUS environment reported that incentives coupled with social influence promoted greater weight loss [190]. In this way, social gaming facilitates the development of intrinsic motivation through the gratification of entertainment, challenge, and competition from game playing and *harnessing the power of others* [191,192]. There have been conflicting reports of the effectiveness of extrinsic incentives and/or penalties to promote health behaviors, depending on the target user region, gender, race, and income [193,194].

Goal setting is an important factor in achieving self-regulatory health behavior. Bandura and associates assigned obese subjects to goal conditions in which they either tracked eating behavior or set subgoals for reducing portions [195]. Within the goal-setting conditions, subjects adopted either weekly or proximal goals for each of 4 time periods during each day. The results demonstrated that setting a higher standard for goals and the adoption of proximal goals resulted in greater weight loss. As demonstrated in the Pagoto et al (2014) study, predetermined proximal goals in combination with Web-based social support improve adherence, support for others, and self-regulation. Research suggests that breaking down goals into subgoals may influence subsequent goal pursuit by reducing goal pursuit because competing subgoals may be perceived as complementary and become substitutes for one another [196].

A Web-based randomized controlled weight loss intervention combined PA and nutrition interventions over a 12-month period [197]. Strategies were guided by SCT theory and included goal setting, self-monitoring, and social support. Participants in the experimental group achieved positive health behavior change (mean z score=+1.34 [*P*<.001] SD units). An interactive Web-based program was developed to set goals relative to the participant’s initial stage of change, revise goals frequently, track behaviors, and deliver graphical feedback. This study demonstrated that interventions can successfully target multiple behaviors simultaneously.

### User-Centered Design

Design objectives for building a community of support for health and wellness should include Web-enabled interaction between the environment and individual choices, which comprise essential components of Bandura’s model of social determinism [198]. Bandura maintained that not only does the environment impact behavior, but human behavior influences the environment. Therefore, health sites should offer tools that support the affective, cohesive, interactive, and social presence needs of the site visitor to increase program engagement [199,200]. An example of a Web-enabled clinical trial, the Enabling Mothers to Prevent Pediatric Obesity Through Web-Based Education and Reciprocal Determinism (EMPOWER) study, focused on encouraging mothers to make changes in the home environment, develop coping skills, form positive expectations, and build self-efficacy [185,201]. The EMPOWER program was delivered in short audiovisual educational presentations, goal planning exercises, tracking worksheets, and a discussion board. Process evaluation data were collected after each session using telephone counseling and Web-based surveys. As predicted by SCT, the intervention resulted in significant increases in child PA, fruit and vegetable consumption, and sugar-free beverage consumption compared with an informational approach. Another study tested the effects of social presence cues (2 staring eyes) on the activation of health-related goals within an ecommerce site [202]. The analysis yielded significant main effects for social presence (*F*_1,218_=5.89; *P*=.016; *d*=0.32) and health goal activation (*F*_1,218_=4.11; *P*=.04; *d*=0.27) on the selection of healthier menu choices compared with the control condition. The combined effects of social presence cues and health-related goal activation produced greater effects on food choices when activated at the same time. Social presence was also associated with the participant perception of success in self-regulatory behavior.

User-centered website design has been successfully used in a chronic disease intervention for patients with type 2 diabetes [203]. In this study, focus group members requested features that included personalized information about their health status, quantified self-tracking tools to monitor progress, and online forums to share their personal experiences. *Personally controlled health management systems* (PCHMSs) have been adopted in many health care organizations to give patients better ways to manage their health. Future studies should identify the best PCHMS tools and evaluate their effectiveness for disease management and prevention. A study on the use of the social and self-reflective features of a PCHMS was designed to support physical and emotional well-being, and frequent use of PCHMS was associated with help-seeking behaviors and increased health care utilization [204]. Although PCHMS tools are common in health care, only 1 trial was located in this search.

In the Self-Help, Exercise and Diet using Information Technology (SHED-IT) study, tailored health communications were specifically designed to address the desired health behavior of adult males and various predictor variables [205,206]. Adherence to the goal setting (beta=−0.3 95% CI −0.6 to −0.1; *P*=.01) and volume of SCT tracking tasks completed (beta=−0.2 95% CI −0.4 to −0.0; *P*=.03) independently predicted weight loss [207]. Message strategies best matched to individual health-related goals increased the impact of functional support. In a meta-analysis of 88 papers on computer health interventions, the use of dynamically tailored interventions gained efficacy over time [208].

### Online Health Communities and Social Networking

A small feasibility trial used a social networking site (Facebook) to promote PA among low active teens who reported medium-to-large changes in PA as measured by accelerometry and self-report [209]. An RCT of college students evaluated the efficacy of a Web-based PA intervention that combined information, self-monitoring, and Web-based social networking strategies in comparison with an instruction-only control [210]. Participants were invited to the Internet Support for Healthy Associations Promoting Exercise (INSHAPE) study website to complete Web-based surveys on their perceived social support for PA (informational, esteem, and companionship subscales), and the Facebook Intensity Scale, a measure of engagement with social networking. The researcher observed and recorded Facebook interactions during the intervention, including comments, discussion forum posts, and affective responses to the comments of others (*like button*). The participation rates in this study were higher than in other published studies. The main effects from the analysis were PA time, esteem, and companionship social support. The authors concluded that real and virtual social connections should be used for group assignment and to match people by profile to encourage PA in future studies.

A mixed-methods study of SparkPeople®, a large online weight loss community, was undertaken to determine how social media frequency predicted perception of Web-based social support for weight loss [211]. The first phase of the study surveyed members for their experience with social support within the community using qualitative analysis of responses to open-ended questions (n=193). Survey respondents were frequent users of email, blogs, and forum discussions. The uses and gratifications for Web-based social support that emerged were informational, emotional, instrumental, appraisal, and network support [212]. The quantitative analysis examined the factor structure for social support from the 7 social media use items (n=187). Principal components analysis of social support items proved to be a 1-dimensional *social media* variable. Social media was a significant predictor of encouragement support (odds ratio [OR] 4.8, 95% CI 1.8 to 12.8; *P*<.001), but not for information or shared experiences support. The authors concluded that members sought encouragement and motivational support for other members, and finding these gratifications was a key determinant to behavioral modification persistence.

The subthemes identified in the study by Hwang et al (2010) included accountability, friendly competition, and humor for a weight loss community [212]. These themes were important motivators in a social media campaign that challenged friends and family to do 1-min core strengthening exercises each day and broadcast individual progress on Twitter (*#PlankADay*) and other social media platforms [213]. Starting out as a friendly challenge between associates, the researchers soon recognized that the message had a spreading network effect. Seizing on this opportunity for research, an Institutional Review Board review was requested and approval was given to begin data collection. The observations of this study are consistent with a framing effect hypothesis of positive messages under a low efficacy condition [214]. The *#PlankADay* message attracted the attention of people who desired core strength but did not take the time to work at gaining core strength. Positive messages lead to more effortful processing of the message. As the challenge was framed in a positive way and tweets contained humor, it is weighed more heavily than a negative message.

### Web-Based Lifestyle Coaching Supports Self-Efficacy

The 4 major sources of self-efficacy beliefs can be transferred to a Web-enabled system: enactive mastery experiences, modeling, social persuasion, and psychological states [215]. A qualitative study of veterans in the Veterans Administration (VA) health system who were cancer survivors revealed many barriers as well as facilitators to their weight management goals [216]. The focus group identified wellness facilitators to boost self-efficacy such as information about their disease, being held accountable for their behaviors, motivational support from others, workout partners, and the ability to visualize healthy changes. Web-based tools enabled the veterans to believe that they can achieve their health goals. The VA health care system pioneered the use of telemedicine and in 1 study compared the efficacy of home TeleHealth monitoring for diabetes management with usual care plus monthly phone calls [217]. Although both groups showed improvement in blood glucose levels, the TeleHealth program results were superior. The TeleHealth program had the advantage of accountability and feedback for behaviors, whereas the monthly phone call with the nurse practitioner provides social presence and feedback. The addition of Web-enabled tools provides the social support that is often lacking in the lives of aging veterans.

Motivational interviewing (MI) is a client-centered counseling approach for eliciting behavior change. A collaborative partnership is developed between coach and client, allowing the participant to discuss goals from the previous week, and problem-solving tools to make goal revisions needed. In a review of Web-based interventions for type 2 diabetes, positive outcomes were associated with using barrier identification, problem solving, and self-monitoring techniques [218]. A randomized, controlled Web-based intervention for depression and stress offered problem solving therapy via email [219]. The goal was to reduce stress and anxiety by providing feedback on exercises that taught problem solving in a structured way. Recently, the use of avatars to deliver health coaching over a Web app was tested, but findings suggest that participants do not readily develop social relationships with avatars and this tool contributed little to the effects of the intervention [220,221].

Enactive learning occurs with a participant observing their own progress through self-monitoring tools, which has been shown to fortify the participant’s self-efficacy beliefs [222]. Bandura suggested that the self-regulation of behavior must be measured against the difficulty of individual obstacles [6]. Over time, the use of Web-enabled tools to monitor diet intake, PA, and other health metrics will provide a rich database to study the effect of past experience, goal setting, and health outcomes. Data from a Web-enabled weight loss intervention for African Americans suggest that early and frequent use of Web-based self-monitoring tools predicts greater weight loss [223]. Web monitoring facilitates positive self-talk by providing evidence of success.

### Social Network Adoption and Participation

The recruitment and retention of network members to support health behavior adoption is another area of research. Social networking is a Web-enabled process that draws people and organizations together. Peers with *experiential similarity* offer support [224]. In an RCT, researchers sought to study the reciprocal learning process (social proof, verbal persuasion, self-monitoring, and frequent feedback) between peers in a Web-based social media intervention [225]. The researchers issued a daily wellness challenge by text or email to 1503 participants, using a small steps approach to both the experimental and control groups. Participants in the intervention group who had access to the well-being challenges were encouraged to recruit others from their social network to join. The measures included the Individual-level Well-Being Assessment and Scoring Method (IWBS), the Interpersonal Support Evaluation List, and intensity of site use [226,227]. After 30 days, the IWBS was significantly greater in the social intervention group than nonsocial participants (µ=9.4 intervention vs 7.0 control group; *P*<.02). Intensity of use was a positive predictor of well-being.

### Implementation of a Theory-Based Framework for Digital Health Intervention

**Figure 2:**
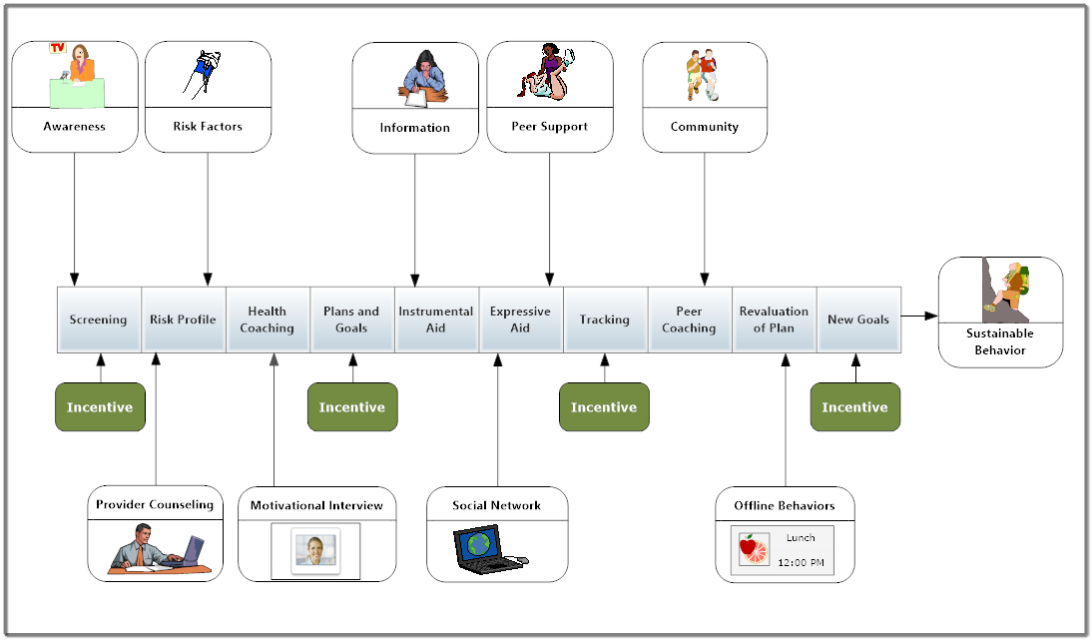
Digital Health Intervention Model. A comprehensive health intervention program integrating social cognitive principles in a web-enabled pathway.

The Digital Health Intervention model (DHIM), shown in Figure 2, integrates theoretical frameworks discussed in this paper and proposes a model of Web-based health promotion based on social cognitive, health behavior, and prospect theories. The DHIM could be applied in various health promotion settings from a worksite wellness to patient-centered medical homes. Researchers could use the results of a health screening questionnaire to populate a risk profile to generate a visual dashboard or navigator. Each contact with the site should have a social presence that recognizes the user as a unique individual and interactions should give the impression that the system is built to help them specifically. As people are slow to adopt new technology when ease of use and perceived utility do not meet user expectations, the technology should be tested for user-friendliness [228]. A confusing interface reduces effectiveness for disease management interventions [229].

*Coaching* can be delivered virtually through delivery of tailored messages, or through phone and chat synchronous communication with call center personnel. An interactive page with games and peer-to-peer connectivity gives the participant support for making behavioral change and opportunities to practice. Plans and goals become part of the dashboard profile available for the member and coach to review. For integrated personal health record systems and patient portals, email access for provider communication is important [230]. The user can seek instrumental aid for informational needs in the form of a video and library database. The development of an intentionally designed social network to support the health goals of the user supplies both instrumental and expressive aid through facilitating self-disclosure and emotional support exchanges between peers [231]. But for stigmatized health issues, such as HIV, informational needs and expressive support may best be met in anonymity rather than within a social networking environment [232]. The Web can enable those with mental health to come out of the shadows and seek treatment as demonstrated by the Web-based program, *Considering Professional Help*, designed to encourage veterans to seek mental health care [233].

Many health-related networks are imbedded with tools that allow the user to upload data from glucose meters, diet trackers, and exercise monitors. These health networks have been shown to positively impact the adoption of health behaviors [19,186]. Behavioral self-monitoring by recording, reporting, and revising action plans are significant predictors of goal attainment, but only when the participant believes that the health goal has motivational value [234]. Therefore, the reward value of the goal is an important factor in motivating behavior change [235].

## Discussion

### Principal Findings

All of the studies reviewed used multimedia informational aids in their health management strategy. Expressive aid was found in 48.6% (54/111) of studies categorized as a strong recommendation. The use of targeted expressive aids was seen in 37.5% (15/40) of the lower quality trials. Only 4 studies included all study categories of tools (informational, expressive, gaming, and tailored content). Serious gaming interventions study design were all quality rated as Level 1 A. Higher quality interventions were more likely to employ 3 or more categories of behavior change techniques within their study designs. Owing to the diversity of health behaviors and treatment protocols analyzed in this review, it is not possible to follow-up with a systematic review of common design elements and their effectiveness at this time.

In the studies reviewed, the relative frequency of Web activities to deliver health intervention universally included informational aids within their design, but 1/4 of them did not tailor or personalize content. Almost half of the interventions did not offer tools to obtain expressive aid, and only 5 of the trials employed serious gaming. This pattern suggests that many health researchers believe that eHealth is primarily an evidence-based informational tool for acquisition and learning of specific health-related skills rather than a *dose* of social influence to encourage health behavior adoption [236].

### Limitations

Many of the studies rejected in this search process were excluded because the authors failed to identify the *a priori* theoretical framework underlying the design strategy or referenced protocols from previous papers which were not theory driven. The risk of selection and publication bias is greater because of the lack of consensus as to publication guidelines that included theory in design considerations during this period. The Cochrane Database of Reviews recently withdrew the publication of a protocol to assess serious gaming health intervention studies with no explanation [237]. Publication guidelines are needed to improve the reporting of clinical trials using serious gaming for future systematic review and analysis. Few studies addressed how their Web-enabled protocol addressed the Health Insurance Portability and Accountability Act security rules or whether privacy was a concern for participants. The potential for innovative technologies such as virtual reality or artificial intelligence to individualize health care is great, but threats to personal health information security may produce distrust among users and health professionals. A systematic review of the literature on website design and interactivity concluded that security elements have an important impact on Web-based health information seeking [238]. These design elements are important patient-reported measures associated with research involvement and should be included in the design of future studies [239]. There is a need for theoretically driven, continuous cohort studies to assess the sustainability of user engagement with tailored digital tools for chronic disease management [240].

### Recommendations

Researchers should define the theory or theories that guide their choices of interventions as well as the desired behaviors that lead to health outcomes. More research is needed to identify the conditions under which media richness and social presence enhance message processing. Creating an environment where social presence is part of the research design may lead to better study retention and greater understanding of Web-based peer-to-peer support. Researchers should consider the addition of a run-in-time phase to their intervention protocols where participant observations can establish the ecological validity of the environment before the intervention. Interactions with social agents, whether human or artificial, are the ultimate tools of social cognition.

### Conclusions

SCTs provide a framework for design, implementation, and evaluation of health intervention programs and has been successfully used in several interventions presented in this paper. Creating Web-based environments where social presence and information richness are used as part of the overall strategy has several theoretical advantages. Web-based health interventions have the potential to act as *sticky* media that sustains the pursuit of desired diet and exercise behaviors long after the initial study phase is over. Using SCT to design digital interventions has been shown to have positive outcomes for weight control, PA, diabetes, mental health, nutrition, and wellness behaviors. The ultimate challenge for health practitioners is to integrate Web-enabled health communication into real-world health care.

## Appendix

Multimedia Appendix 1 Preferred Reporting Items for Systematic Reviews and Meta-Analyses flow diagram.

Multimedia Appendix 2 Study characteristics. Internet-based intervention characteristics.

## Abbreviations

CINAHL: Cumulative Index to Nursing and Allied Health Literature
CONSORT-EHEALTH: Consolidated Standards of Reporting Trials of Electronic and Mobile HEalth Applications and onLine TeleHealth
DHIM: Digital Health Intervention Model
eHealth: electronic health
EMPOWER: Enabling Mothers to Prevent Pediatric Obesity Through Web-Based Education and Reciprocal Determinism
GUS: games used seriously
iCBT: internet cognitive behavioral therapy
INSHAPE: Internet Support for Healthy Associations Promoting Exercise
IWBS: Individual-level Well-Being Assessment and Scoring Method
JBI: Joanna Briggs Institute
mHealth: mobile health
MI: motivational interviewing
PA: physical activity
PCHMS: Personally Controlled Health Management Systems
PRISMA-ScR: Preferred Reporting Items for Systematic reviews and Meta-Analyses extension for Scoping Reviews
RCT: randomized controlled trial
SCT: social cognitive theory
SHED-IT: Self-Help, Exercise and Diet using Information Technology
VA: Veterans Administration
