# Supplementary figures and images for: Social Cognitive Theories and Electronic Health Design: Scoping Review
Source: JMIR Hum Factors. 2019 Jul 19;6(3):e11544. doi: 10.2196/11544 (PMC6676794; doi:10.2196/11544)

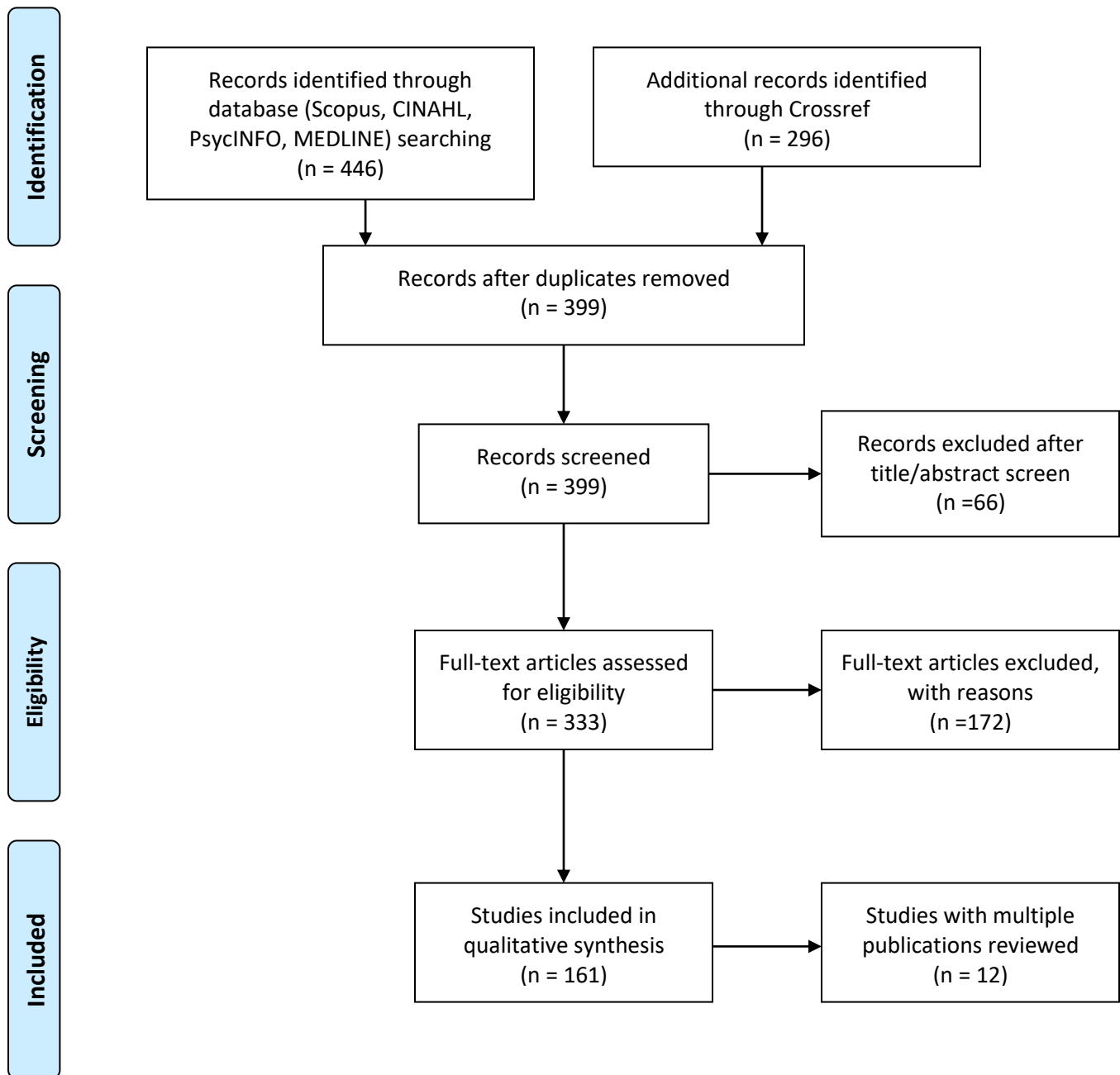

Supplement: Multimedia Appendix 1 [file humanfactors_v6i3e11544_app1.pdf]
