# Supplementary material for: Social Cognitive Theories and Electronic Health Design: Scoping Review
Source: JMIR Hum Factors. 2019 Jul 19;6(3):e11544. doi: 10.2196/11544 (PMC6676794; doi:10.2196/11544)
Supplement: Multimedia Appendix 2 [file humanfactors_v6i3e11544_app2.pdf]

Multimedia Appendix 2. Study characteristics. Internet-based intervention characteristics.

Study	Theory	Quality	Informational aid	Expressive aid	Gaming	Tailored
Agricola et al (2014) [37]	TPB[a]	Level 3.e B	X	—	—	X
Allam et al (2015) [38]	iCBT[b]	Level 1.c A	X	X	X	X
Anderson et al (2013) [39]	SRT[c]	Level 1.c A	X	X	—	—
Antypas et al (2014) [40]	RFT[d]	Level 1.c B	X	X	—	—
Bannink et al (2014) [41]	CBT[e]	Level 1.c A	X	X	—	X
Becker et al (2014) [42]	BCT[f]	Level 1.c B	X	—	—	X
Bendtsen et al (2015) [43]	BCT[f]	Level 1.c B	X	—	—	X
Bendtsen et al (2012) [44]	BCT[f]	Level 1.c B	X	—	—	X
Bewick et al (2013) [45]	BCT[f]	Level 1.c A	X	—	—	X
Bock et al (2013) [46]	BCT[f]/TTM[g]/I-change[h]	Level 1.c A	X	—	—	X
Bolier et al (2013) [47]	BCT[f]	Level 1.c A	X	X	—	X
Boots et al (2016, 2018) [48, 49]	BCT[f]	Level 1.c A	X	X	—	X
Børøsund et al (2014) [50]	BCT[f]	Level 1.c A	X	—	—	—
Bossen et al (2013) [51]	BCT[f]	Level 1.c A	X	X	—	X

Brendryen et al (2014) [52]	CBT[e]	Level 1.c A	X	—	—	—
Brindal et al (2012) [53]	BCT[f]	Level 1.c B	X	X	—	X
Bul et al (2016) [54]	BCT[f]	Level 1.c A	X	—	X	X
Burckhardt et al (2015) [55]	BCT[f]	Level 1.c B	X	X	—	X
Camerini et al (2012) [56]	BCT[f]	Level 1.c B	X	X	—	X
Carlson et al (2012) [57]	BCT[f]	Level 1.c A	X	X	—	X
Carter et al (2013) [58]	BCT[f]	Level 1.c A	X	—	—	X
Cavallo et al (2012) [59]	BCT[f]	Level 1.c B	X	—	—	X
Choi et al (2014) [60]	BCT[f]	Level 1.c A	X	—	—	X
Christensen et al (2014) [61]	BCT[f]/psychoeducation	Level 1.c B	X	—	—	X
Clarke et al (2016) [62]	BCT[f]/SCT[i]	Level 3.e A	X	X	—	X
Collins et al (2012, 2013) [63, 64]	Social influence theory	Level 1.c A	X	—	—	X
Compernolle et al (2015) [65]	CBT[e]	Level 1.c A	X	X		X
Cook et al (2014) [66]	CBT[e]	Level 1.c A	X	—	—	—
Cremers et al (2015) [67]	CBT[e]	Level 1.c A	X	X	—	X
Cremers et al (2014) [68]	CBT[e]	Level 1.b A	X	X	—	X
Cristancho-Lacroix et al (2015) [69]	CBT[e]	Level 1.c B	X	—	—	—

Cunningham et al (2012) [70]	CBT[e]	Level 1.c A	X	X	—	X
Damholdt et al (2016) [71]	CBT[e]	Level 1.c A	X	X	—	X
De Cocker et al (2012) [72]	SRT[j]/CCM[k]	Level 1.c B	X	X	—	X
Delisle et al (2015), Nystrom (2017) [73,74]	CCM[k]	Level 1.c A	X	—	—	X
de Josselin et al (2014) [75]	SRT[j]/CCM[k]	Level 1.c A	X	X	—	X
Dennison et al (2014) [76]	CCM[k]/behavior change	Level 1.c B	X			X
Devi et al (2014) [77]	CCM[k]/SCT[i]/behavior change	Level 1.c A	X	X	—	X
Direito et al (2015) [78]	CBT[e]/IPT[l]	Level 1.c B	X	—	—	X
Dobson et al (2016, 2018) [79, 80]	CBT[e]	Level 1.c A	X	X	—	X
Donker et al (2013) [81]	CBT[e]	Level 1.c B	X	X	—	—
Drozd et al (2013) [82]	CBT[e]	Level 1.c A	X		—	—
Duncan et al (2014) [83]	CBT[e]	Level 1.c A	X	X	—	X
Ebert et al (2013) [84]	CBT[e]	Level 1.c B	X	—	—	X
Elfeddali et al (2012) [85]	GAP[m]	Level 1.c A	X	X	—	—
Emmons et al (2013) [86]	Cognitive therapy	Level 1.c A	X	—	—	X
Eysenbach et al (2015) [87]	TPB[a]/psychosocial well-being	Level 1.c A	X	X	—	X
Fjeldsoe et al (2012, 2015) [88,89]	CTF[n]	Level 1.c A	X	X	—	X

Frederix et al (2015) [90,91]	CTF[n]	Level 1.c A	X	—	—	X
Friederichs et al (2014)[92,93]	CBT[e]	Level 1.c A	X	—	—	—
Geraedts et al (2014) [94]	CBT[e]	Level 1.c A	X	X	—	X
Greaney et al (2012) [95]	CBT[e]	Level 1.c A	X	—	—	X
Greenwood et al (2015) [96]	CBT[e]	Level 1.c A	X	—	—	X
Gustafson et al (2012) [97]	CBT[e]/SCT[i]	Level 1.c B	X	X	—	X
Hansen et al (2012) [98]	CBT[e]/social support	Level 1.c A	X	X	—	X
Hardcastle et al (2012) [99]	TPB[a], SDT[o], TTM[g]	Level 1.c A	X	X	—	X
Haug et al (2013) [100]	CBT[e]	Level 1.c A	X	X	—	X
Hausmann et al (2014) [101]	Forced self-disclosure/social	Level 1.c A	X	X	—	X
Heber et al (2013, 2016) [102,103]	Transactional stress model, iSMI[p]	Level 1.c A	X	X	—	X
Høifødt et al (2013)[104]	CBT[e]	Level 1.c B	X	—	—	X
Imanaka et al (2013) [105]	Health behavioral theory	Level 1.c B	X	—	—	X
Irvine et al (2013) [106]	CBT[e]/TPB[a]	Level 1.c A	X	—	—	X
Irvine et al (2015) [107]	TPB[a]	Level 1.c A	X	—	—	X
Jordan et al (2015) [108]	I-Change model	Level 1.d B	X	—	—	X
Karhula et al (2015) [109]	SCT[i]	Level 1.c B	X	—	—	—

Kass et al (2014) [110]	SLT[q]	Level 1.c B	X	—	—	X
Kelders et al (2014) [111]	ACT[r]/SLT[q]	Level 1.c A	X	—	—	—
Khosropour et al (2013) [112]	TPB[a]/adherence	Level 1.c A	X	—	—	—
Kim et al (2015) [113]	SLT[q]	Level 1.c B	X	—	—	X
Kirwan et al (2013) [114]	TPB[a]	Level 1.c B	X	—	—	—
Knaevelsrud et al (2015) [115]	CBT[e]	Level 1.c A	X	—	—	X
Knowlden et al (2015) [116]	SCT[i]/reciprocal determinism	Level 1.c A	X	—	—	—
Kok et al (2014) [117]	CBT[e]	Level 1.c A	X	—	—	—
Kolodziejczyk et al (2013) [118]	SCT[i]/CT[s]/SET[t]	Level 2.d B	X	—	—	X
Kramer et al (2014) [119]	BCT[f]/SFBT[u]	Level 1.c A	X	—	—	X
Krein et al (2013) [120]	Knowledge	Level 1.c A	X	—	—	
Kristjánsdóttir et al (2013) [121]	iCBT[v]	Level 1.c B	X	X	—	X
Krukowski et al (2013) [122]	SRT[c]	Level 1.c A	X	X	—	X
Leahey, et al (2014) [123]	MBCT[w]/SCT[i]	Level 3.e A	X	X	X	X
Levy et al (2015) [124]	CCM[k]	Level 1.c A	X	—	—	X
Maher et al (2015) [29]	Self-efficacy	Level 1.c A	X	X	X	X
Mak et al (2015) [125]	Positive psychology	Level 1.c A	X	—	—	—

Manicavasagar et al (2014) [126]	Positive psychology	Level 1.c A	X	X	—	—
Marsaux et al (2015) [127]	Positive psychology	Level 1.c A	X	—	—	—
Marsaux et al (2016) [128]	Gene-based personalized/TTM[g]/	Level 1.c A	X	X	—	X
Martorella et al (2012) [129]	BCT[f]/ELM[x]	Level 1.c B	X	—	—	X
Mattila et al (2013) [130]	CBT[e]/problem solving	Level 1.c A	X	—	—	X
McClure et al (2013) [131]	Procrastination-health model	Level 1.c A	X	—	—	—
Mehring et al (2016) [132]	CBT[e]	Level 1.c B	X	X	—	X
Meyer et al (2015) [133]	CBT[e]/positive psychology	Level 1.c A	X	X	—	X
Milgrom et al (2016) [134]	Psychoeducational	Level 1.c A	X	—	—	X
Mira et al (2014) [135]	TPB[a]/SCT[i]	Level 1.c A	X	X	—	X
Morgan et al (2014) [136]	SCT[i]	Level 1.c A	X	—	—	X
Mori et al (2014) [137]	SCT[i]	Level 1.c B	X	—	—	X
Morris et al (2015) [138]	SCT[i]	Level 1.c A	X	X	—	X
Mouthaan et al (2013) [139]	SCT[i]	Level 1.c B	X	X	—	X
Müller et al (2016) [140]	SCT	Level 1.c A	X	X	—	X
Newcombe et al (2012) [141]	SCT[i]	Level 1.c B	X	X	—	X
Nobis et al (2013) [142]	SCT[i]	Level 1.c A	X	X	—	X

Partridge et al (2015) [143]	SCT[i]	Level 1.c A	X	X	—	X
Pham et al (2016) [144]	SCT[i]/CBT[e]	Level 1.c A	X	—	—	X
Piette et al (2015) [145]	SRT[c]	Level 1.c A	X	X	—	X
Pimmer et al (2013) [146]	SCT[i]/SDT[o]	Level 1.c A	X	—	—	X
Plaete et al (2016) [147]	SCT[i]/SRT[c]	Level 1.c A	X	—	—	X
Proudfoot et al (2013) [148]	CBT[e]	Level 1.c A	X	X	—	X
Powell et al (2012) [149]	SCT[i]/TTM[g]	Level 1.c A	X	—	—	X
Rabbi et al (2015) [150]	SDT[o]	Level 1.c A	X	X	—	X
Ramadas et al (2011, 2015) [151,152]	SDT[o]	Level 1.c A	X	X	—	X
Riva et al (2014) [153]	SDT[o]/self-efficacy	Level 1.c A	X	X	X	X
Robinson et al (2014, 2016) [154,155]	SDT[o]/SCT[i]	Level 1.c A	X	X	—	X
Rooke et al (2013) [156]	SDT[o]/health values	Level 1.c A	X	—	—	X
Salazar et al (2014) [157]	SCT[i]	Level 1.c A	X	X	—	X
Samaan et al (2013) [158]	SCT[i]	Level 1.c A	X	X	—	—
Schaller et al (2016) [159]	SCT[i]	Level 1.c B	X	—	—	X
Schaub et al (2012) [160]	SCTi	Level 1.c A	X	—	—	X
Schulz et al (2014) [161]	SCT[i]/i-Change[h]	Level 1.c A	X	—	—	X

Smit et al (2012) [162]	SCT/i-Change[h]	Level 1.c B	X	—	—	—
Solomon et al (2012) [163]	TPB[a]	Level 1.c A	X	—	—	—
Soureti et al (2011) [164]	SCT[i]/GAPm	Level 1.c A	X	—	—	X
Springvloet et al (2015) [165]	SCT[i]/precaution adoption/GAP[m]	Level 1.c B	X	X	—	X
Steinberg et al (2013) [166]	SCT[i]	Level 1.c A	X	—	—	—
Steinberg et al (2014) [167]	SCT[i]	Level 1.c A	X	X	—	X
Tait et al (2015) [168]	SCT[i]/bystander educational model	Level 1.c A	X	—	—	—
Tapper et al (2014) [169]	SCT[i]/TTM[g]	Level 1.c A	X	—	—	X
Tensil et al (2013) [170]	SCT[i]/precaution adoption process	Level 1.c B	X	—	—	X
ter Huurne et al (2015) [171]	Social influences/SCT[i]	Level 1.c A	X	X	—	X
Torbjørnsen et al (2014) [172]	Social norms theory/SMT[y]	Level 1.c B	X	—	—	—
Trompetter et al (2015) [173]	SCT[i]/engagement	Level 1.c A	X	X	—	X
Turner-McGrievy et al (2014) [174]	SFBT[u]	Level 1.c A	X	X	—	X
Ünlü Ince et al (2013) [175]	Supportive accountability	Level 1.c B	X	X	—	X
van Ballegooijen et al (2013) [176]	TPB[a]	Level 1.c B	X	—	—	X
van der Weegen et al (2015) [177]	TBP[a]/fun theory	Level 1.c A	X	X	—	X

van der Wulp et al (2014) [178]	CBT[e]	Level 1.c B	X	X	—	X
van der Zanden et al (2012) [179]	SCT[i]/SMT[y]	Level 1.c A	X	X	—	X
van Gaalen et al (2013) [180]	SRT[c]	Level 1.c A	X	X	—	X
van Genugten et al (2014) [181]	SRT[c]	Level 1.c A	X	—	—	X
van Straten et al (2008) [182]	Knowledge/patient empowerment	Level 1.c A	X	X	—	X
Vandelanotte et al (2012) [183]	SCT[i]	Level 1.c A	X	—	—	X
Volker et al (2015) [184]	CBT[e]	Level 1.c A	X	—	—	X
Voogt et al (2014) [185]	CBT[e]/motivation enhancement	Level 1.c A	X	—	—	—
Vroege et al (2014) [186]	TPB[a]	Level 1.c A	X	—	—	—
Vuorinen et al (2014) [187]	TPB[a]/TTM[g]	Level 1.c A	X	—	—	X
Walthouwer et al (2015) [188]	TPB[a]/TTM[g]	Level 1.c A	X	—	—	X
Wang et al (2013) [189]	TTM[g]	Level 1.c A	X	X	—	X
Watson et al (2012) [190]	TTM[g]	Level 1.c A	X	—	—	X
Watson et al (2015) [191]	TTM[g]	Level 1.c A	X	—	—	X
Wayne et al (2015) [192]	TTM[g]/BCT[f]/social support	Level 1.c A	X	X	—	X
Weymann et al (2015) [193]	BCT[f]/SMT[y]/empowe rment	Level 1.c A	X	—	—	X
Whittaker et al (2012) [194]	CBT[e]/self-efficacy beliefs	Level 1.c A	X	X	—	X

Wijsman et al (2013) [27]	TTM[g]	Level 1.c A	X	—	—	X
Wójcicki et al (2014) [195]	CBT[e]/ART[z]	Level 1.c B	X	X	—	X
Ybarra et al (2012) [196]	SCT[i]/social norms	Level 1.c B	X	—	—	X

Study characteristic: X=present; —=absent

---

[a] theory of planned behavior
[b] internet cognitive behavioral therapy
[c] self-regulation theory
[d] relational frame theory
[e] cognitive behavioral therapy
[f] behavioral control theory
[g] transtheoretical model
[h] i-Change model
[i] social cognitive theory
[j] self-regulation theory
[k] chronic care model
[l] interpersonal psychotherapy
[m] goal-setting
[n] cognitive therapy framework
[o] social determination theory
[p] internet-based stress management intervention
[q] social learning theory
[r] acceptance and commitment therapy
[s] control theory
[t] social ecological theory
[u] solution-focused brief therapy
[v] internet cognitive behavioral therapy
[w] mindfulness-based cognitive therapy
[x] elaboration likelihood model
[y] self-management theory
[zz] affect regulation training

References

27. Wijsman CA, Westendorp RG, Verhagen EA, Catt M, Slagboom PE, de Craen AJ, et al. Effects of a web-based intervention on physical activity and metabolism in older adults: randomized controlled trial. J Med Internet Res. 2013 Nov 6;15(11):e233. PMID: 24195965. doi: 10.2196/jmir.2843.

37. Agricola E, Pandolfi E, Gonfiantini MV, Gesualdo F, Romano M, Carloni E, et al. A cohort study of a tailored web intervention for preconception care. BMC medical informatics and decision making. 2014 Apr 15;14:33. PMID: 24731520. doi: 10.1186/1472-6947-14-33.

38. Allam A, Kostova Z, Nakamoto K, Schulz PJ. The effect of social support features and gamification on a Web-based intervention for rheumatoid arthritis patients: randomized controlled trial. J Med Internet Res. 2015;17(1):e14. PMID: 25574939. doi: 10.2196/jmir.3510.

39. Andersen LL, Sundstrup E, Boysen M, Jakobsen MD, Mortensen OS, Persson R. Cardiovascular health effects of internet-based encouragements to do daily workplace stair-walks: randomized controlled trial. J Med Internet Res. 2013 Jun 21;15(6):e127. PMID: 23793032. doi: 10.2196/jmir.2340.

40. Antypas K, Wangberg SC. An Internet- and mobile-based tailored intervention to enhance maintenance of physical activity after cardiac rehabilitation: short-term results of a randomized controlled trial. J Med Internet Res. 2014 Mar 11;16(3):e77. PMID: 24618349. doi: 10.2196/jmir.3132.

41. Bannink R, Broeren S, Joosten-van Zwanenburg E, van As E, van de Looij-Jansen P, Raat H. Effectiveness of a Web-based tailored intervention (E-health4Uth) and consultation to promote adolescents' health: randomized controlled trial. J Med Internet Res. 2014 May 30;16(5):e143. PMID: 24878521. doi: 10.2196/jmir.3163.

42. Becker J, Haug S, Sullivan R, Schaub MP. Effectiveness of different Web-based interventions to prepare co-smokers of cigarettes and cannabis for double cessation: a three-arm randomized controlled trial. J Med Internet Res. 2014 Dec 05;16(12):e273. PMID: 25486674. doi: 10.2196/jmir.3246.

43. Bendtsen P, Bendtsen M, Karlsson N, White IR, McCambridge J. Online Alcohol Assessment and Feedback for Hazardous and Harmful Drinkers: Findings From the AMADEUS-2 Randomized Controlled Trial of Routine Practice in Swedish Universities. J Med Internet Res. 2015 Jul 09;17(7):e170. PMID: 26159179. doi: 10.2196/jmir.4020.

44. Bendtsen P, McCambridge J, Bendtsen M, Karlsson N, Nilsen P. Effectiveness of a proactive mail-based alcohol Internet intervention for university students: dismantling the assessment and feedback components in a randomized controlled trial. J Med Internet Res. 2012 Oct 31;14(5):e142. PMID: 23113955. doi: 10.2196/jmir.2062.

45. Bewick BM, West RM, Barkham M, Mulhern B, Marlow R, Traviss G, et al. The effectiveness of a Web-based personalized feedback and social norms alcohol intervention on United Kingdom university students: randomized controlled trial. J Med Internet Res. 2013 Jul 24;15(7):e137. PMID: 23883616. doi: 10.2196/jmir.2581.

46. Bock B, Heron K, Jennings E, Morrow K, Cobb V, Magee J, et al. A Text Message Delivered Smoking Cessation Intervention: The Initial Trial of TXT-2-Quit: Randomized Controlled Trial. JMIR mHealth and uHealth. 2013 Jul 30;1(2):e17. PMID: 25098502. doi: 10.2196/mhealth.2522.

47. Bolier L, Haverman M, Kramer J, Westerhof GJ, Riper H, Walburg JA, et al. An Internet-based intervention to promote mental fitness for mildly depressed adults: randomized controlled trial. J Med Internet Res. 2013 Sep 16;15(9):e200. PMID: 24041479. doi: 10.2196/jmir.2603.

48. Boots LM, de Vugt ME, Kempen GI, Verhey FR. Effectiveness of the blended care self-management program "Partner in Balance" for early-stage dementia caregivers: study protocol

for a randomized controlled trial. Trials. 2016 May 4;17(1):231. PMID: 27142676. doi: 10.1186/s13063-016-1351-z.

49. Boots LM, de Vugt ME, Kempen GI, Verhey FR. Effectiveness of a Blended Care Self-Management Program for Caregivers of People With Early-Stage Dementia (Partner in Balance): Randomized Controlled Trial. J Med Internet Res. 2018 Jul 13;20(7):e10017. PMID: 30006327. doi: 10.2196/10017.

50. Borosund E, Cvancarova M, Moore SM, Ekstedt M, Ruland CM. Comparing effects in regular practice of e-communication and Web-based self-management support among breast cancer patients: preliminary results from a randomized controlled trial. J Med Internet Res. 2014 Dec 18;16(12):e295. PMID: 25525672. doi: 10.2196/jmir.3348.

51. Bossen D, Veenhof C, Van Beek KE, Spreeuwenberg PM, Dekker J, De Bakker DH. Effectiveness of a web-based physical activity intervention in patients with knee and/or hip osteoarthritis: randomized controlled trial. J Med Internet Res. 2013 Nov 22;15(11):e257. PMID: 24269911. doi: 10.2196/jmir.2662.

52. Brendryen H, Lund IO, Johansen AB, Riksheim M, Nesvag S, Duckert F. Balance--a pragmatic randomized controlled trial of an online intensive self-help alcohol intervention. Addiction. 2014 Feb;109(2):218-26. PMID: 24134709. doi: 10.1111/add.12383.

53. Brindal E, Freyne J, Saunders I, Berkovsky S, Smith G, Noakes M. Features predicting weight loss in overweight or obese participants in a web-based intervention: randomized trial. J Med Internet Res. 2012 Dec 12;14(6):e173. PMID: 23234759. doi: 10.2196/jmir.2156.

54. Bul KC, Kato PM, Van der Oord S, Danckaerts M, Vreeke LJ, Willems A, et al. Behavioral Outcome Effects of Serious Gaming as an Adjunct to Treatment for Children With Attention-Deficit/Hyperactivity Disorder: A Randomized Controlled Trial. J Med Internet Res. 2016 Feb 16;18(2):e26. PMID: 26883052. doi: 10.2196/jmir.5173.

55. Burckhardt R, Manicavasagar V, Batterham PJ, Miller LM, Talbot E, Lum A. A Web-Based Adolescent Positive Psychology Program in Schools: Randomized Controlled Trial. J Med Internet Res. 2015 Jul 28;17(7):e187. PMID: 26220564. doi: 10.2196/jmir.4329.

56. Camerini L, Schulz PJ. Effects of functional interactivity on patients' knowledge, empowerment, and health outcomes: an experimental model-driven evaluation of a web-based intervention. J Med Internet Res. 2012 Jul 18;14(4):e105. PMID: 22810046. doi: 10.2196/jmir.1953.

57. Carlson JA, Sallis JF, Ramirez ER, Patrick K, Norman GJ. Physical activity and dietary behavior change in Internet-based weight loss interventions: comparing two multiple-behavior change indices. Preventive medicine. 2012 Jan;54(1):50-4. PMID: 22085706. doi: 10.1016/j.ypmed.2011.10.018.

58. Carter MC, Burley VJ, Nykjaer C, Cade JE. Adherence to a smartphone application for weight loss compared to website and paper diary: pilot randomized controlled trial. J Med Internet Res. 2013 Apr 15;15(4):e32. PMID: 23587561. doi: 10.2196/jmir.2283.

59. Cavallo DN, Tate DF, Ries AV, Brown JD, DeVellis RF, Ammerman AS. A social media-based physical activity intervention: a randomized controlled trial. Am J Prev Med. 2012 Nov;43(5):527-32. PMID: 23079176. doi: 10.1016/j.amepre.2012.07.019.

60. Choi SH, Waltje AH, Ronis DL, Noonan D, Hong O, Richardson CR, et al. Web-enhanced tobacco tactics with telephone support versus 1-800-QUIT-NOW telephone line intervention for operating engineers: randomized controlled trial. J Med Internet Res. 2014 Nov 20;16(11):e255. PMID: 25447467. doi: 10.2196/jmir.3375.

61. Christensen H, Batterham P, Mackinnon A, Griffiths KM, Kalia Hehir K, Kenardy J, et al. Prevention of generalized anxiety disorder using a web intervention, iChill: randomized controlled trial. J Med Internet Res. 2014 Sep 02;16(9):e199. PMID: 25270886. doi: 10.2196/jmir.3507.

62. Clarke J, Proudfoot J, Ma H. Mobile Phone and Web-based Cognitive Behavior Therapy for Depressive Symptoms and Mental Health Comorbidities in People Living With Diabetes: Results of a Feasibility Study. JMIR mental health. 2016 May 31;3(2):e23. PMID: 27245948. doi: 10.2196/mental.5131.

63. Collins CE, Morgan PJ, Jones P, Fletcher K, Martin J, Aguiar EJ, et al. A 12-week commercial web-based weight-loss program for overweight and obese adults: randomized controlled trial comparing basic versus enhanced features. J Med Internet Res. 2012 Apr 25;14(2):e57. PMID: 22555246. doi: 10.2196/jmir.1980.

64. Collins CE, Morgan PJ, Hutchesson MJ, Callister R. Efficacy of standard versus enhanced features in a Web-based commercial weight-loss program for obese adults, part 2: randomized controlled trial. J Med Internet Res. 2013 Jul 22;15(7):e140. PMID: 23876832. doi: 10.2196/jmir.2626.

65. Compernolle S, Vandelanotte C, Cardon G, De Bourdeaudhuij I, De Cocker K. Effectiveness of a web-based, computer-tailored, pedometer-based physical activity intervention for adults: a cluster randomized controlled trial. J Med Internet Res. 2015 Feb 09;17(2):e38. PMID: 25665498. doi: 10.2196/jmir.3402.

66. Cook TL, De Bourdeaudhuij I, Maes L, Haerens L, Grammatikaki E, Widhalm K, et al. Moderators of the effectiveness of a web-based tailored intervention promoting physical activity in adolescents: the HELENA Activ-O-Meter. J Sch Health. 2014 Apr;84(4):256-66. PMID: 24617909. doi: 10.1111/josh.12140.

67. Cremers HP, Mercken L, Candel M, de Vries H, Oenema A. A Web-based, computer-tailored smoking prevention program to prevent children from starting to smoke after transferring to secondary school: randomized controlled trial. J Med Internet Res. 2015 Mar 09;17(3):e59. PMID: 25759248. doi: 10.2196/jmir.3794.

68. Cremers HP, Mercken L, Crutzen R, Willems P, de Vries H, Oenema A. Do email and mobile phone prompts stimulate primary school children to reuse an Internet-delivered smoking prevention intervention? J Med Internet Res. 2014 Mar 18;16(3):e86. PMID: 24642082. doi: 10.2196/jmir.3069.

69. Cristancho-Lacroix V, Wrobel J, Cantegreil-Kallen I, Dub T, Rouquette A, Rigaud AS. A web-based psychoeducational program for informal caregivers of patients with Alzheimer's disease: a pilot randomized controlled trial. J Med Internet Res. 2015 May 12;17(5):e117. PMID: 25967983. doi: 10.2196/jmir.3717.

70. Cunningham JA. Comparison of two internet-based interventions for problem drinkers: randomized controlled trial. J Med Internet Res. 2012 Aug 1;14(4):e107. PMID: 22954459. doi: 10.2196/jmir.2090.

71. Damholdt MF, Mehlsen M, O'Toole MS, Andreasen RK, Pedersen AD, Zachariae R. Web-based cognitive training for breast cancer survivors with cognitive complaints-a randomized controlled trial. Psycho-oncology. 2016 Nov;25(11):1293-300. PMID: 26763774. doi: 10.1002/pon.4058.

72. De Cocker K, Spittaels H, Cardon G, De Bourdeaudhuij I, Vandelanotte C. Web-based, computer-tailored, pedometer-based physical activity advice: development, dissemination through general practice, acceptability, and preliminary efficacy in a randomized controlled trial. J Med Internet Res. 2012 Apr 24;14(2):e53. PMID: 22532102. doi: 10.2196/jmir.1959.

73. Delisle C, Sandin S, Forsum E, Henriksson H, Trolle-Lagerros Y, Larsson C, et al. A web- and mobile phone-based intervention to prevent obesity in 4-year-olds (MINISTOP): a population-based randomized controlled trial. BMC public health. 2015 Feb 07;15:95. PMID: 25886009. doi: 10.1186/s12889-015-1444-8.

74. Nystrom CD, Sandin S, Henriksson P, Henriksson H, Trolle-Lagerros Y, Larsson C, et al. Mobile-based intervention intended to stop obesity in preschool-aged children: the MINISTOP randomized controlled trial. The American journal of clinical nutrition. 2017 Jun;105(6):1327-35. PMID: 28446496. doi: 10.3945/ajcn.116.150995.

75. de Josselin de Jong S, Candel M, Segaar D, Cremers HP, de Vries H. Efficacy of a Web-based computer-tailored smoking prevention intervention for Dutch adolescents: randomized controlled trial. J Med Internet Res. 2014;16(3):e82. PMID: 24657434. doi: 10.2196/jmir.2469.
